# A Green μQuEChERS/HPLC-PDA Method for Phytochemical Profiling and Bioactivity Assessment of Tomato (*Solanum lycopersicum*) Varieties

**DOI:** 10.3390/foods15122110

**Published:** 2026-06-11

**Authors:** Carina Fernandes, Gonçalo Jasmins, Gonçalo N. Martins, Paula C. Castilho, José S. Câmara, Rosa Perestrelo

**Affiliations:** 1 CQM—Centro de Química da Madeira, Universidade da Madeira, Campus da Penteada, 9020-105 Funchal, Portugal; carinacst2004@gmail.com (C.F.); goncalo.jasmins@staff.uma.pt (G.J.); goncalo.martins@staff.uma.pt (G.N.M.); pcastilho@staff.uma.pt (P.C.C.); 2 Departamento de Química, Faculdade de Ciências Exatas e Engenharia, Universidade da Madeira, Campus da Penteada, 9020-105 Funchal, Portugal

**Keywords:** tomato, lipophilic profile, antioxidant activity, μQuEChERS, UHPLC-PDA

## Abstract

Tomato (*Solanum lycopersicum*) is one of the most extensively cultivated crops worldwide and a recognized dietary-rich source of phytochemicals associated with cardioprotective, antioxidant, antidiabetic, anti-inflammatory, anticancer, antimicrobial, and anti-aging properties. This study provides a comprehensive comparative assessment of the lipophilic composition, total phenolic content (TPC), and antioxidant capacity of six *Solanum* varieties, namely Roma, Kumato, Globe, and Vine (*S. lycopersicum* L.), Cherry (*S. lycopersicum* var. cerasiforme), and Tamarillo (*S. betaceum*), using a validated μQuEChERS/HPLC-PDA analytical approach combined with complementary in vitro antioxidant assays. The optimized analytical method exhibited robust analytical performance, with strong linearity (R^2^ ≥ 0.993), high sensitivity and selectivity, satisfactory precision (%RSD < 20%), and acceptable recoveries (78–118%), confirming its suitability for routine profiling of lipophilic compounds in complex matrices. Significant differences (*p* < 0.05) were observed among varieties, highlighting the strong role of genotype in modulating lipophilic phytochemical accumulation. Kumato and Cherry exhibited the highest levels of lycopene, β-carotene, and tocopherols, whereas Globe consistently exhibited the lowest lipophilic antioxidant content. In vitro assays identified Vine with the greatest TPC (290 µgGAE/g dw) and antioxidant activity (1603 µgTE/g dw), while Globe recorded the lowest values (194 µgGAE/g dw and 1395 µgTE/g dw, respectively). Hierarchical clustering analysis further corroborated these findings, revealing three chemically distinct clusters: Vine and Tamarillo associated with superior TPC and antioxidant activity; Cherry and Kumato characterized by elevated carotenoid and tocopherol content; and Globe and Roma distinguished by comparatively lower nutritional quality across all parameters assessed. These results demonstrate that the validated μQuEChERS/HPLC-PDA method is a reliable, sensitive, and efficient tool for comparative phytochemical profiling of tomato varieties. The observed compositional differences may contribute to future studies focused on nutritional evaluation, traceability, and authenticity assessment of tomato-derived products.

## 1. Introduction

Tomato (*Solanum lycopersicum* L.) is one of the most widely cultivated crops worldwide, with annual production exceeding 190 million tons in 2023 [1]. Its global relevance stems not only from its high consumption as a fresh fruit and as a raw material for processed products, but also from its economic and cultural importance in the agri-food sector [2]. Tomato-based products such as pastes, sauces, purees, juices, and ketchups represent a significant fraction of the food market, contributing to the income of producers and processors across Europe and the Mediterranean basin [2,3,4]. Alongside its economic significance, the tomato is an excellent source of health-promoting phytochemicals, including carotenoids (e.g., lycopene and β-carotene), tocopherols (vitamin E isomers), phenolic compounds, flavonoids, and anthocyanins. These phytochemicals contribute to the fruit’s nutritional and functional value, being associated with antioxidant, anti-inflammatory, cardioprotective, neuroprotective and anticancer effects [5,6,7,8].

The compositional richness of tomatoes makes them attractive not only to consumers and nutritionists, but also to the food industry. However, this high demand has also made tomato and its derived products particularly vulnerable to fraudulent practices. Adulteration, mislabeling of varieties, and the substitution of high-quality raw materials with lower-quality counterparts have been reported in different segments of the tomato supply chain. For instance, processed tomato products can be adulterated with cheapeners, coloring agents, or even extracts from other fruits and vegetables to mimic the characteristic color and taste of genuine tomatoes. Misrepresentation of geographical origin or variety identity is another form of fraud with both economic, legal and regulatory implications. Such practices not only mislead consumers but also undermine fair competition and may compromise nutritional quality and food safety [9,10,11,12].

Ensuring authenticity in tomatoes and tomato-derived products requires the development of robust analytical methods capable of generating reliable phytochemical fingerprints. Traditional approaches to extract and quantify phytochemicals in tomatoes include Soxhlet extraction [13], solid phase extraction (SPE) [2], ultrasound-assisted extraction [4], pressurized liquid extraction [11,14], and solid–liquid extraction (SLE) [2,3,15], which, although widely used, are associated with several drawbacks. They are typically time-consuming, require large amounts of solvent and sample, and may lack reproducibility when applied to large sample sets. Moreover, from a sustainability perspective, extraction procedures with high solvent consumption and long extraction times are not aligned with the principles of green analytical chemistry. The demand for rapid, eco-friendly, and sensitive alternatives with reduced solvent and sample consumption has increased in recent years. In this framework, QuEChERS (Quick, Easy, Cheap, Effective, Rugged, Safe) emerged as a gold-standard extraction procedure. Initially developed for pesticide residue analysis, QuEChERS has since been adapted to meet the demands of a wide range of food, environmental and clinical applications. The μQuEChERS approach minimizes sample and solvent requirements while maintaining high extraction efficiency and reproducibility. This microscale procedure consists of green analytical chemistry principles, as it reduces chemical waste and analytical costs, while remaining sufficiently versatile for complex matrices such as tomato [16,17]. Moreover, when combined with high-performance liquid chromatography coupled to photodiode array detection (HPLC-PDA), μQuEChERS provides a powerful platform for the simultaneous extraction, identification, and quantification of key phytochemicals. UHPLC-PDA allows multi-wavelength monitoring of analytes, which is essential for discriminating carotenoids, tocopherols, and phenolic compounds that absorb different spectral regions. This capability ensures accurate separation and detection of compounds that are often present at low concentrations and may otherwise overlap in complex chromatograms. Furthermore, PDA detection facilitates spectral confirmation of peak identity, thereby strengthening the method’s selectivity.

The integration of μQuEChERS with UHPLC-PDA may contribute to future authenticity and traceability studies by generating reproducible phytochemical profiles associated with different tomato varieties. Carotenoids and tocopherols can serve as chemical markers of varietal and geographical origin, as their concentration and distribution are strongly influenced by cultivar genetics, ripening stage, environmental conditions, and cultivation practices [3,18]. For example, lycopene content is a major determinant of tomato color. It is widely used to distinguish between red, yellow, and orange varieties, whereas tocopherol profiles may provide complementary information on nutritional value and authenticity. Establishing reliable phytochemical fingerprints can thus support traceability systems, authenticate products with protected designation of origin (PDO) or protected geographical indication (PGI), and detect adulteration or substitution in processed goods [19,20].

Beyond authentication, phytochemical profiling also supports the evaluation of bioactivity, reinforcing the nutritional and functional claims of tomato products. Antioxidant assays such as ABTS and DPPH, in combination with spectrophotometric assays for total phenolics, flavonoids, carotenoids, and anthocyanins, provide complementary evidence of the health-promoting potential of different varieties. These bioactivity assessments are essential not only for consumer health perspectives but also for positioning tomato varieties in value-added markets where nutritional and functional quality are key aspects [12,15].

Despite the growing interest in green extraction techniques and phytochemical profiling of tomatoes, there is still a lack of integrated approaches combining miniaturized extraction procedures with comprehensive lipophilic and bioactivity assessment across multiple varieties. The current study aimed to optimize and validate a green μQuEChERS/HPLC-PDA strategy for comparing phytochemical profiling and antioxidant assessment of different tomato varieties. The method was optimized and validated to ensure analytical robustness. Then, it was applied to quantify key carotenoids (lycopene, β-carotene) and vitamin E isomers (α-, β-, and γ-tocopherol). In parallel, complementary in vitro assays were employed to evaluate total phenolic, flavonoid, carotenoid, and anthocyanin content, as well as antioxidant activity. This study aimed to assess the integration of the tomato’s phytochemical profile and in vitro assays’ data with hierarchical cluster analysis (HCA) as a tool to compare, characterize and classify different tomato varieties.

## 2. Materials and Methods

### 2.1. Reagents

All reagents were of analytical grade. HPLC-grade methanol, ethanol, and hexane were from Fischer Scientific (Loughborough, UK). Gallic acid (purity ≥  99%), quercetin (99%), trolox (98%), and β-carotene (≥97%) were purchased from Fluka (Buchs, Switzerland), while lycopene (≥85%), and tocopherols (mixture of α, β, Δ, and γ-tocopherols, FG) were obtained from Sigma-Aldrich (St. Louis, MO, USA). These standards were used to identify and quantify lipophilic compounds in tomato extracts. Stock solutions of each standard were prepared at a concentration of 500 mg/L and stored at −80 °C. 2,2′-Azinobis-(3-ethylbenzothiazoline-6-sulfonic acid (ABTS^●^, 98%), Folin–Ciocalteu reagent (FR, 2N), sodium chloride (NaCl), sodium carbonate (Na_2_CO_3_), 2,2-diphenyl-1-picrylhydrazyl (DPPH^●^, 90%), anhydrous magnesium sulfate (MgSO_4_), sodium potassium (KCl), sodium acetate (CH_3_COONa), sodium citrate tribasic dihydrate (C_6_H_5_Na_3_O_7_ · 2H_2_O), hydrochloric acid (HCl, 37% *v*/*v*), sodium citrate dibasic sesquihydrate (C_6_H_5_Na_2_O_7_ · 1.5 H_2_O), butylated hydroxytoluene (BHT, ≥99%, used as stabilizer) and acetyl chloride were obtained from Sigma-Aldrich (St. Louis, MO, USA). Aluminum chloride (AlCl_3_, 98%) and potassium persulfate (K_2_S_2_O_8_, 99%) were purchased from Riedel-de Haën^®^ (Seelze, Germany). Ultrapure water (18 MΩ·cm) from a Milli-Q system (Millipore, Milford, MA, USA) was used for all aqueous solutions.

### 2.2. Sample Collection

Six tomato varieties were selected for analysis: Roma, Kumato, Globe, and Vine (*Solanum lycopersicum* L.), Cherry (*S. lycopersicum* var. cerasiforme), and Tamarillo tomato (*S. betaceum*), all purchased from a local market on Madeira Island. Tamarillo was included as a complementary *Solanum* variety due to its recognized nutritional relevance and commercial availability. However, comparisons between *S. betaceum* and *S. lycopersicum* varieties should be interpreted cautiously due to inherent interspecies metabolic differences.

Ten individual fruits of every variety were used as biological replicates to ensure adequate representation, with sampling restricted to a fully ripe fruit, at a stage that is ready for consumption. Before processing, non-edible parts such as pedicels and sepals were cautiously removed. The fruits were then rinsed with water, manually sliced, and homogenized to generate a single composite sample per variety. After that, the tomatoes were lyophilised (Telstar, Cryodos, Madrid, Spain) for eight hours, then ground in a lab mill (Grindomix GM200, Rech, Germany) to obtain a fine, homogeneous powder and kept at −80 °C until analysis. This exploratory study used composite sampling to obtain representative phytochemical profiles for each variety and minimize individual fruit variability. Therefore, environmental variables (e.g., cultivation practices, geographical origin, seasonal variability) and intra-varietal biological variability were not independently evaluated.

### 2.3. μQuEChERS Procedure to Extract Lipophilic Compounds

The μQuEChERS procedure was adapted from the technique reported by Abreu et al. [21] and by Melfi et al. [22]. Briefly, 0.3 g of lyophilised tomato and 0.3 g of a partitioning salt mixture (maintaining the original QuEChERS ratio, 4 MgSO_4_:1 NaCl:1 C_6_H_5_Na_3_O_7_ · 2H_2_O:0.5 C_6_H_5_Na_2_O_7_ · 1.5 H_2_O) were measured into a 5 mL screw-capped centrifuge tube. Then, 1 mL of 50:30:20 *v*/*v*/*v* of hexane, acetone, and ethanol solution, containing 0.5% BHT (*w*/*v*), was added. The tube was vortexed for 30 s, sonicated in ultrasonic bath (Bransonic 2510) for 5 min, and then centrifuged for 5 min at 5000 rpm (centrifuge: SIGMA 1–7, St. Louis, MO, USA, maximum capacity 6 × 15 mL, maximum RCF 6153× *g*), which resulted in phase separation between the apolar (hexane-rich) and polar (acetone/ethanol-rich) fractions. The polar phase was collected for subsequent in vitro assays using a UV-Vis spectrophotometer (Lambda 25, PerkinElmer, Waltham, MA, USA), while the upper apolar phase was collected and filtered through a 0.22 μm PTFE filter membrane for subsequent identification and quantification of lipophilic compounds in HPLC-PDA, and total carotenoid content (TC) determination by UV-Vis spectrophotometer. The extraction procedure for each tomato variety was performed in triplicate.

### 2.4. HPLC-PDA Conditions

The identification and quantification of carotenoids (lycopene, β-carotene) and vitamin E isoforms (α-, β-, and γ-tocopherol) were performed by HPLC-PDA using the method of Melfi et al. [22], with minor modifications. Analyses were carried out on a Dionex UltiMate 3000 system (Thermo Scientific, Vacaville, CA, USA) using a Phenomex Gemini C18 column (5 µm, 250 × 3.0 mm i.d.). Chromatographic separation was performed isocratically with acetonitrile/methanol/hexane (90/8/2, %*v*/*v*/*v*) containing 0.1% acetic acid at 0.5 mL/min, using a 10 µL volume injection, and column temperature of 35 °C. The extracts were screened (200–880 nm) and detected at 298 nm (tocopherols), 450 nm (carotenoids), and 472 nm (lycopene). The tocopherols and carotenoids were identified by retention times against pure standards and confirmed by comparison with UV/Vis spectra reported in the literature.

### 2.5. Method Validation

μQuEChERS/HPLC-PDA quantitative analytical method for the determination of lipophilic compounds in tomato extracts was validated in terms of selectivity, linearity, sensitivity, accuracy, and precision. Selectivity was confirmed by the comparison of target analyte retention times (RTs), PDA spectra, and peak purity with that of reference standards. Linearity was established by calibration curves (n = 7) of 2.6–467 mg/L by least-squares regression against the range of α-, β-, δ-tocopherol, lycopene, and β-carotene variability. Sensitivity was estimated by the determination of LOD (S/N = 3) and LOQ (S/N = 10) at the lowest level of each analyte. Precision was evaluated in terms of repeatability (intra-day, n = 6) and reproducibility (inter-day, n = 30 over five days), and was expressed as relative standard deviation (%RSD, relative standard deviation) at three levels of concentration (low, medium, high) within the calibration range. Accuracy was also presented as percentage recovery using Roma tomato spikes at three levels of concentration (low, medium, high) within the calibration range.

### 2.6. Bioactivity Assessment Using In Vitro Assays

The total phenolics (TPC), total flavonoids (TFC), total anthocyanins (TAC), total carotenoids (TC) and antioxidant capacity (ABTS, DPPH) of tomato extracts obtained by µQuEChERS were determined spectrophotometrically, with all assays performed in triplicate. TPC and TFC were quantified by the Folin–Ciocalteu and AlCl_3_ colorimetric assays, respectively, following Abreu et al.’s [21] protocol, and the results were expressed as µg gallic acid equivalents (GAEs) and µg quercetin equivalents (QEs) per g dry weight (dw), respectively. TAC was calculated by the pH differential assay [21] and TC using the protocol of Popescu et al. [23], and the results were expressed as µg cyanidin-3-glucoside equivalents (C3GEs) and µg β-carotene equivalents (βCEs) per g dw, respectively. Antioxidant activity was assessed by DPPH (2,2-diphenyl-1-picrylhydrazyl) and ABTS (2,2′-azino-bis(3-ethylbenzothiazoline-6-sulfonic acid) assays [21], with results expressed as µg Trolox equivalents (TEs) per g dw.

### 2.7. Statistical Analysis

All experiments were conducted in triplicate, and data were analyzed with MetaboAnalyst 6.0 [24] using one-way ANOVA followed by Tukey’s test (*p* < 0.05). Analytical measurements were performed in triplicate for each composite sample. Therefore, the statistical analysis reflects analytical reproducibility rather than biological replication among independent variety batches. Multivariate analysis, including hierarchical cluster analysis (HCA), based on Euclidean distance and Ward’s method, was performed to reveal clustering patterns for the characterization of the tomato extracts.

## 3. Results and Discussion

### 3.1. μQuEChERS/HPLC-PDA Validation

The μQuEChERS/HPLC-PDA method was validated for linearity, selectivity, sensitivity, precision, and accuracy to ensure its suitability for the quantitative determination of tocopherols and carotenoids in tomato extracts. Table 1 summarizes the analytical parameters obtained during method validation.

All analytes exhibited excellent linearity (R^2^ = 0.993–0.999), demonstrating a strong correlation between concentration and detector response. The LOD ranged from 0.03 to 0.59 mg/L, while LOQ ranged from 0.09 to 1.97 mg/L. It was observed that tocopherols showed higher sensitivity than carotenoids, which was consistent with their stronger absorbance at 298 nm and more favorable detection conditions. Nevertheless, the sensitivity achieved for carotenoids was suitable for their determination in the different tomato extracts.

Precision was evaluated at three concentration levels (low, medium, and high) and expressed as %RSD. Intra-day precision ranged from 0.88% to 5.57%, while inter-day precision ranged from 2.14% to 8.50%. Tocopherols exhibited lower variability than carotenoids, whereas β-carotene showed slightly higher RSD values, particularly at low concentrations, likely due to its susceptibility to oxidation and isomerization.

Accuracy was assessed at three concentration levels (low, medium, and high) yielding recoveries between 78% and 118%. Tocopherols exhibited consistent recoveries (86–118%), with β-tocopherol showing slightly elevated values (>110%), indicative of minor matrix effects. Lycopene exhibited satisfactory recoveries (85–96%), whereas β-carotene showed lower recovery at high concentration (78%), which may be attributed to degradation or analytical losses.

The validated μQuEChERS/HPLC-PDA method demonstrated analytical performance comparable to previously reported HPLC methodologies for simultaneous determination of tocopherols and carotenoids in tomato varieties, with satisfactory linearity, sensitivity, selectivity, precision and accuracy [2,22]. Irakli et al. [2] optimized and validated a conventional SLE and SPE followed by HPLC-DAD/FLD analysis using a C30 column for simultaneous tocopherols and carotenoids in tomatoes. The method achieved excellent linearity (R^2^ = 0.9964–0.9997), recoveries (91.7–103.4%), intra-day precision ranging from 1.4 to 6.6%, and inter-day precision between 3.0 and 9.4% RSD. Although highly sensitive and robust, the method required multiple extraction steps, solvent evaporation, reconstitution procedures, and employed chlorinated solvents such as dichloromethane. The present μQuEChERS/HPLC-PDA strategy offers important practical advantages for routine phytochemical screening and high-throughput analysis. Although some recovery values were slightly broader than those reported in highly optimized conventional HPLC protocols, the analytical performance remained within acceptable validation criteria for complex food matrices, while considerably enhancing method simplicity and analytical sustainability. Moreover, compared with previously reported methods based on conventional SLE or SPE, the proposed μQuEChERS approach offers practical advantages, including miniaturization, reduced solvent consumption and sample amount, simplified sample preparation, and increased analytical throughput.

### 3.2. Quantification of Tocopherols and Carotenoids in Solanum Varieties

The concentration (µg/g dw) of tocopherols and carotenoids in tomato extracts is shown in the following figures. Significant differences among six *Solanum* varieties were observed for all individual lipophilic compounds (*p* < 0.05) using Tukey’s HSD post hoc analysis, highlighting the strong influence of genotype on their accumulation.

**Figure 1:**
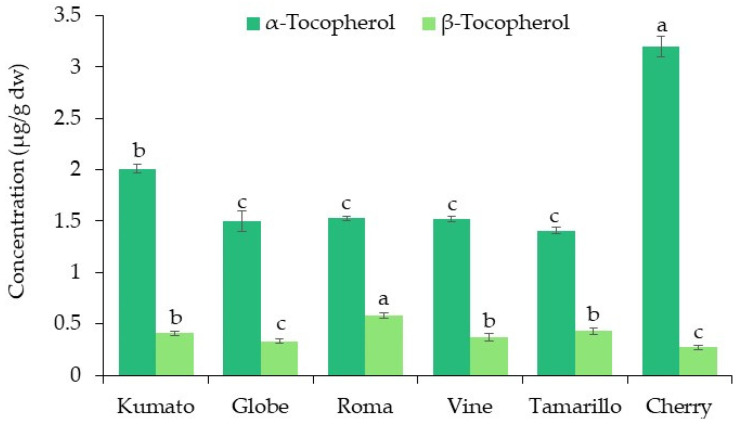
Tocopherols (α and β) content across the six *Solanum* varieties studied. Different lowercase letters indicate significant differences among varieties (Tukey HSD, *p* < 0.05).

The tocopherols concentration (Figure 1) varied significantly among the six *Solanum* varieties analyzed (*p* < 0.05), α-tocopherol being the predominant form in all samples.

α-Tocopherol concentrations ranged from 1.41 ± 0.03 µg/g dw (Tamarilho) to 3.2 ± 0.1 µg/g dw (Cherry), with Cherry exhibiting significantly higher levels compared to all other varieties (*p* < 0.05) (grouped into classes b and c without statistically significant differences). These findings suggest that genotypic variation plays a determining role in α-tocopherol accumulation, consistent with previous reports indicating that vitamin E biosynthesis in tomato is strongly influenced by cultivar-specific genetic factors. The α-tocopherol concentrations observed in this study are within the range reported for fresh tomatoes, varying from 1.7 to 4.3 µg/g depending on cultivar and growing conditions [2,25]. β-Tocopherol was detected at considerably lower concentrations, ranging from 0.27 ± 0.02 µg/g dw (Cherry) to 0.58 ± 0.03 µg/g dw (Roma). Notably, Cherry exhibited the highest α-tocopherol but the lowest β-tocopherol content, suggesting varietal differences in tocopherol distribution patterns that warrant further metabolic investigation.

The total tocopherol content followed the order Cherry > Kumato > Roma > Vine > Tamarillo > Globe, identifying Cherry as the variety with the greatest vitamin E potential among *S. lycopersicum* varieties, and Roma as the richest source of β-tocopherol across all species evaluated. These findings underscore the nutritional value of tomato as a dietary source of vitamin E and emphasize the importance of variety selection for maximizing tocopherol intake.

**Figure 2:**
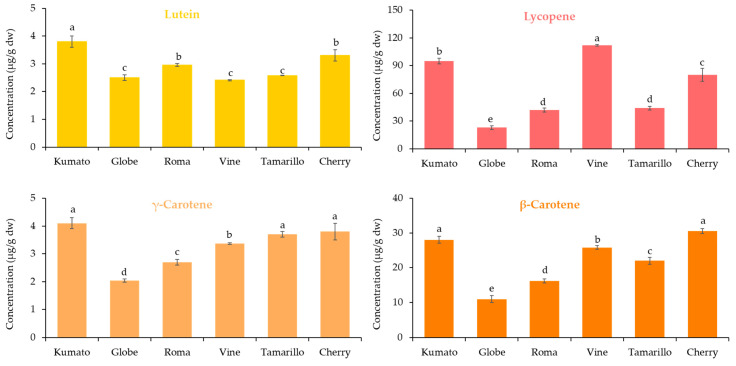
Carotenoid profile in the six *Solanum* varieties studied. Different lowercase letters indicate significant differences among tomato varieties (Tukey HSD, *p* < 0.05).

The carotenoid composition of the six *Solanum* varieties differed significantly across all compounds analyzed (*p* < 0.05), reflecting the strong influence of genotype and species on the lipophilic phytochemical profile (Figure 2).

**Figure 3:**
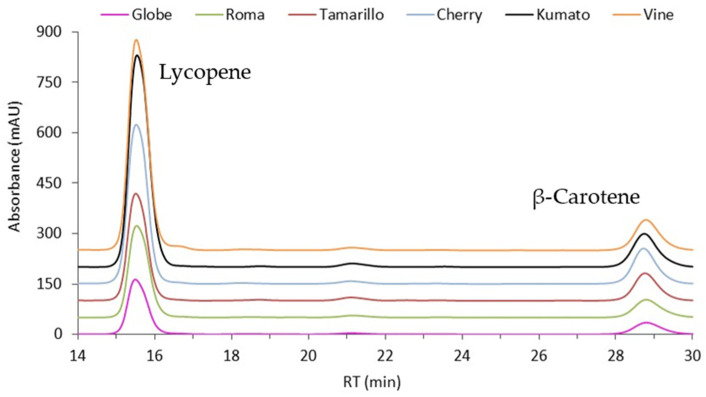
Overlap of typical chromatogram (λ = 450 nm) of lycopene and β-carotene in the different tomato extracts analyzed by the μQuEChERS/HPLC-PDA methodology.

Total carotenoid content in the six *Solanum* varieties ranged from 38.5 to 143.2 µg/g dw, with lycopene accounting for an average of 68% of the total carotenoid fraction. Vine had the highest lycopene concentration (112 µg/g dw), while Globe had the lowest (23 µg/g dw), as can be seen in Figure 3.

Moreover, no significant difference was observed in lycopene concentration between Roma and Tamarillo varieties (*p* > 0.05). These findings agree with the literature, in which lycopene concentrations in fresh tomatoes typically range from 36 to 250 µg/g dw, depending on cultivar, ripeness, and environmental conditions [2,4,25]. The relatively high lycopene content observed in Vine, Kumato, and Cherry varieties suggests enhanced antioxidant potential, as this lipophilic compound is broadly recognized for its strong singlet oxygen quenching ability and its association with reduced risk of chronic diseases [26]. β-carotene was the second most abundant carotenoid detected across all samples, with the highest levels observed in Cherry (30.6 µg/g dw) and the lowest in Globe (11 µg/g dw). Nevertheless, no statistical difference in β-carotene concentration (*p* > 0.05) was observed between Cherry and Kumato varieties. These concentrations are slightly higher than those commonly reported for conventional tomato varieties (4 to 17 µg/g dw), which may indicate varietal specificity or differences in cultivation conditions [2]. γ-Carotene and lutein were present at moderate concentration, with Kumato and Cherry varieties exhibiting higher levels, reinforcing their superior antioxidant profiles.

### 3.3. In Vitro Assays to Assess the Bioactivity of Tomato Extracts

The phytochemical composition (TPC, TFC, TAC, TC) and antioxidant activity (ABTS, DPPH) of bioactive-rich extracts from the six *Solanum* varieties investigated were evaluated using six in vitro assays. Data were subjected to analysis of variance (ANOVA), and a subsequent Tukey test to identify significant differences (*p* < 0.05) among *Solanum* varieties (Table 2). Significant differences (*p* < 0.05) among the tomato varieties were observed regarding their phytochemical composition and antioxidant activity, highlighting the strong influence of genotype on the accumulation of bioactive compounds [3].

The TPC in six *Solanum* varieties ranged from 194 to 290 µgGAE/g dw, corresponding to Globe and Vine varieties, respectively. However, no significant differences in TPC were observed among Vine, Kumato and Cherry varieties (*p* > 0.05), indicating comparable phenolic levels. The higher TPC level suggests greater potential for radical scavenging activity, although this relationship is not strictly linear across all samples. On the other hand, Globe (194 µgGAE/g), Roma (198 µgGAE/g) and Tamarillo (217 µgGAE/g) showed significantly lower TPC levels (*p* < 0.05). These findings agree with those reported in the literature for fresh tomatoes, with TPC values ranging between 150 and 400 µgGAE/g depending on cultivar and ripening stage [12,27,28].

The highest TFC content was observed for Tamarilho (167 µg QE/g dw); however, this value was not significantly different from those of Cherry and Kumato (*p* > 0.05), as shown in Table 2. Although Vine exhibited the highest TPC, its TFC is significantly lower (*p* < 0.05), suggesting a phenolic profile dominated by non-flavonoid compounds (e.g., hydroxycinnamic acids), which are known to be abundant in tomatoes [15]. Additionally, no significant differences in TFC were observed among Globe, Roma and Vine (*p* > 0.05).

TAC levels were relatively low (20–34 µgC3GE/g dw), as expected for commercial tomato varieties. Kumato and Cherry exhibited significantly higher TAC compared to other varieties (*p* < 0.05). While tomatoes are not typically rich in anthocyanins, certain genotypes and pigmented varieties can accumulate measurable levels that contribute to their antioxidant capacity [29].

TC showed the greatest variability, ranging from 263 to 760 µgβCE/g dw, with Kumato and Cherry exhibiting the highest content, while Globe displayed the lowest values (*p* < 0.05). Additionally, no significant difference in TC content was observed between Roma and Tamarillo varieties. This finding agrees with previous studies, showing that dark and pigmented tomato varieties typically accumulate higher carotenoid levels, particularly lycopene and β-carotene [30].

Antioxidant activity determined by ABTS and DPPH assays revealed significant differences among the six *Solanum* varieties (*p* < 0.05). ABTS assays yielded higher values (1395 to 1603 µgTE/g dw) than DPPH assays (459–548 µgTE/g dw), consistent with the broader reactivity of ABTS toward both hydrophilic and lipophilic antioxidants [31]. The Vine variety exhibited the highest antioxidant activity in both assays, suggesting synergistic contributions of diverse phytochemical classes, while Globe showed the lowest values. The observed variability among varieties is consistent with previous studies demonstrating that genotype, cultivation conditions, and maturity stage significantly influence phytochemical composition [32,33].

**Figure 4:**
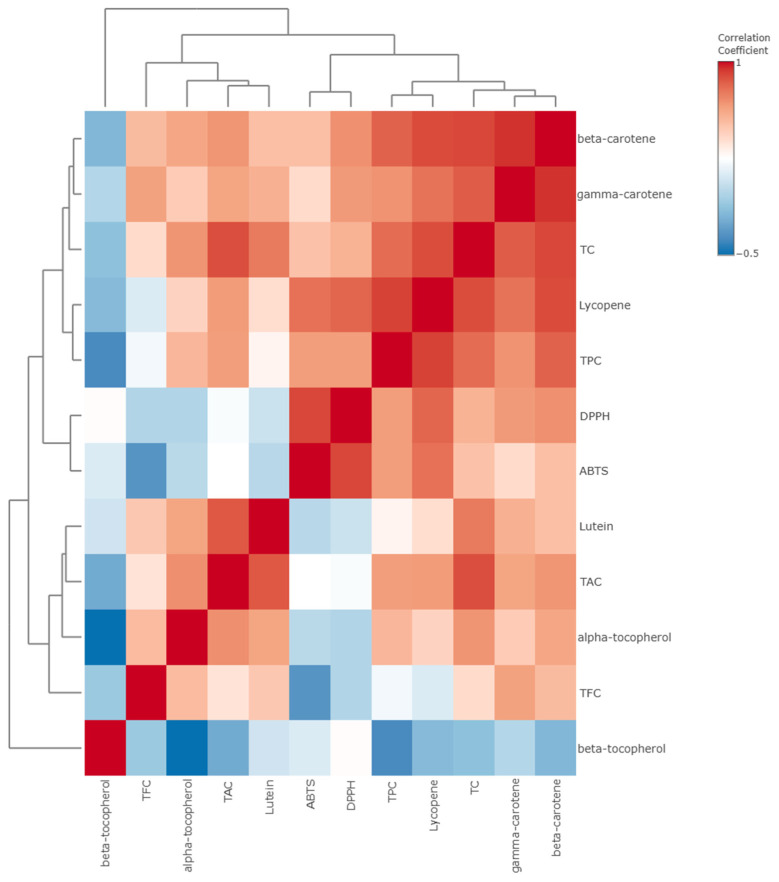
Pearson correlation heatmap shows the relationships between phytochemical composition (TPC, TFC, TAC, TC), lipophilic compounds (tocopherols and carotenoids), and antioxidant activity (ABTS and DPPH) in tomato varieties. Positive correlations are indicated by warmer tones and negative correlations by cooler tones.

The Pearson correlation heatmap (Figure 4) highlights strong positive associations between TC and individual carotenoids, particularly lycopene (r = 0.879) and β-carotene (r = 0.896), confirming their major contribution to carotenoid accumulation. TPC showed moderate correlation with antioxidant activity (r = 0.645), while stronger associations were observed for carotenoids, particularly lycopene (r = 0.908). However, these correlations should be interpreted as associative rather than causal, as antioxidant activity likely results from synergistic contributions of multiple phytochemical families. Antioxidant activity (ABTS and DPPH) displayed strong and moderate associations with carotenoids, whereas tocopherols exhibited weak or negative correlations, indicating a limited contribution to antioxidant capacity. These findings provide new insights into the relative importance of lipophilic versus hydrophilic antioxidants in determining the nutritional quality of tomato varieties. Although individual phenolic compounds were not characterized, previous studies have identified hydroxycinnamic acids (e.g., chlorogenic, caffeic, p-coumaric, and ferulic acids) and flavonoids (e.g., rutin, quercetin derivatives) as major tomato phenolics [34]. These compounds likely contributed to the observed antioxidant activity, although their specific roles require confirmation by HPLC-DAD-MS or LC-MS/MS analysis.

### 3.4. Hierarchical Clustering Analysis

**Figure 5:**
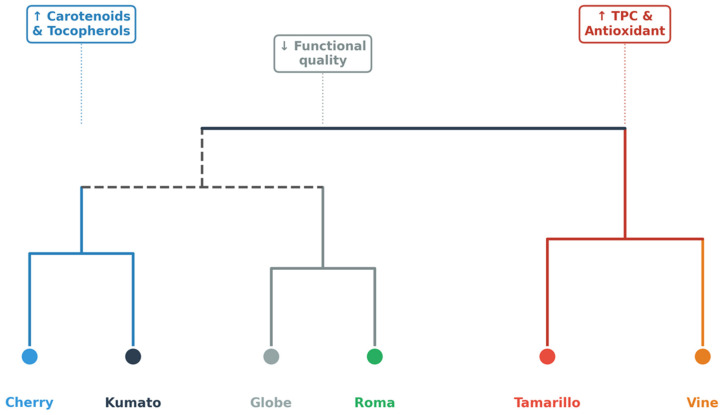
Hierarchical clustering analysis of antioxidant-related parameters (TPC, TFC, TC, TAC), radical scavenging activities (ABTS and DPPH), and lipophilic compounds (lutein, tocopherols, and carotenoids) across different tomato varieties (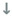 Decrease; 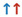 Increase). Data was standardized before analysis, and clustering was performed using Euclidean distance and Ward’s linkage approach. Color scale represents relative abundance, with red indicating higher values and blue indicating lower value.

The hierarchical clustering analysis revealed a clear and consistent grouping of samples according to tomato variety (Figure 5), highlighting the strong influence of genotype on the overall biochemical and antioxidant profile. Moreover, all biological replicates clustered tightly within their respective variety groups, demonstrating a high intra-group similarity and confirming the reproducibility and robustness of the analytical approach. This clustering pattern suggests that the measured variables were suitable to reveal relative phytochemical similarities and differences among the analyzed tomato varieties under the experimental conditions employed.

Hierarchical clustering analysis grouped the six *Solanum* varieties into three statistically distinct clusters reflecting their overall phytochemical similarity.

Cluster 1 (Cherry and Kumato) was defined by elevated carotenoid and tocopherol concentrations, characterizing these varieties as preferential sources of lipophilic antioxidants. Cluster 2 (Globe and Roma) presented the lowest nutritional quality scores across the compound classes assessed, suggesting a comparatively reduced antioxidant potential when all variables are considered simultaneously. Cluster 3 (Tamarillo and Vine) was clearly separated from the remaining varieties by the longest branch length in the dendrogram, indicating the greatest phytochemical distinctiveness, driven by superior total phenolic content and antioxidant activity. The segregation of Tamarillo (*S. betaceum*) alongside the *S. lycopersicum* cultivar Vine within this cluster is particularly noteworthy, as it demonstrates that inter-species divergence in phenolic metabolism can override intra-species varietal differences. Collectively, the HCA results reinforce a nutritional gradient across the Solanum varieties studied and support the use of multivariate chemometric approaches to discriminate cultivars based on their bioactive compound profiles.

## 4. Conclusions

This study demonstrated that tomato variety significantly influences phytochemical composition and antioxidant capacity, with marked genotype-dependent differences observed among the six *Solanum* varieties evaluated. The μQuEChERS/HPLC-PDA method proved to be a reliable, rapid, and environmentally friendly approach for the determination of lipophilic phytochemicals, enabling effective discrimination of tomato varieties based on their carotenoid and tocopherol profiles. Among the varieties analyzed, Vine, Kumato, and Cherry exhibited the most favorable phytochemical and antioxidant profiles, whereas Globe consistently showed the lowest values. Correlation and hierarchical clustering analyses further confirmed the strong contribution of carotenoids, particularly lycopene, to the overall antioxidant capacity of tomato extracts.

Although a positive association between TPC and antioxidant activity was observed, TPC values should be interpreted with caution because the Folin–Ciocalteu assay is not specific to phenolic compounds and may also respond to other reducing substances naturally present in tomatoes, which may affect the total phenolic content. Furthermore, although individual phenolics were not characterized in this study, tomatoes are known to contain hydroxycinnamic acids (e.g., chlorogenic, caffeic, p-coumaric, ferulic acids) and flavonoids (e.g., rutin, quercetin derivatives), which may contribute to their antioxidant properties.

The limitations of this study are the use of samples from a single commercial source and the absence of targeted phenolic characterization by LC-MS/MS. Future studies should integrate comprehensive phenolic profiling with bioactivity assessment to better elucidate the contribution of specific phytochemicals to tomato antioxidant potential and support nutritional and traceability applications.

## Figures and Tables

**Table 1:** μQuEChERS/HPLC-PDA method validation.

RT (min)	Compounds	λ_max_ (nm)	Linear Range (mg/L)	R^2^	LOD (mg/L)	LOQ (mg/L)	Precision (% RSD)			Accuracy (%)
							Intra-Day		Inter-Day	
8.88	δ-Tocopherol	298	2.6–118	0.998	0.44	1.45	LL	2.04	4.58	90 ± 6
							ML	1.57	3.89	86 ± 1
							HL	0.88	2.14	97 ± 1
9.99	β-Tocopherol	298	2.6–118	0.999	0.03	0.09	LL	1.98	2.89	118 ± 1
							ML	2.46	3.57	112 ± 2
							HL	1.87	4.18	114 ± 1
11.3	α-Tocopherol	298	2.6–118	0.996	0.23	0.76	LL	2.35	7.97	87 ± 3
							ML	2.70	5.69	90 ± 4
							HL	1.51	3.28	96 ± 8
15.4	Lycopene	472	10–467	0.993	0.59	1.97	LL	3.17	6.74	96 ± 1
							ML	1.27	3.28	89 ± 3
							HL	1.31	5.37	85 ± 2
28.8	β-Carotene	450	10–467	0.996	0.33	1.1	LL	5.57	8.50	89 ± 3
							ML	3.79	6.07	107 ± 1
							HL	1.78	4.21	78 ± 5

RT—retention time; LOD—limit of detection; LOQ—Limit of quantification.

**Table 2:** Phytochemical-related parameters (TPC, TFC, TC, TAC) and radical scavenging activities (ABTS and DPPH) using in vitro assays.

Tomatoes	TPC (µgGAE/g)	TFC (µgQE/g)	TAC (µgC3GE/g)	TC (µgβCE/g)	ABTS (µgTE/g)	DPPH (µgTE/g)
Kumato	268 ± 21 ^a^	151 ± 9 ^a^	34 ± 1 ^a^	760 ± 18 ^a^	1497 ± 18 ^b^	515 ± 2 ^b^
Globe	194 ± 7 ^b^	119 ± 1 ^b^	21 ± 2 ^b^	263 ± 2 ^e^	1395 ± 13 ^c^	459 ± 1 ^d^
Roma	198 ± 5 ^b^	124 ± 6 ^b^	22 ± 2 ^b^	399 ± 4 ^d^	1505 ± 17 ^b^	510 ± 1 ^b^
Vine	290 ± 11 ^a^	113 ± 2 ^b^	23 ± 1 ^b^	529 ± 4 ^c^	1603 ± 13 ^a^	548 ± 2 ^a^
Tamarillo	217 ± 15 ^b^	167 ± 2 ^a^	20 ± 1 ^b^	375 ± 9 ^d^	1440 ± 7 ^c^	510 ± 1 ^b^
Cherry	277 ± 12 ^a^	166 ± 5 ^a^	29.3 ± 0.2 ^a^	656 ± 18 ^b^	1469 ± 20 ^b^	500 ± 4^c^

Different letters in each column indicate statistically significant differences according to Tukey’s HSD post hoc test following one-way ANOVA (*p* < 0.05) for each assay.

## Data Availability

The original contributions presented in the study are included in the article. Further inquiries can be directed to the corresponding authors.

## Abbreviations

The following abbreviations are used in this manuscript:

ABTS	2,2’-azino-bis(3-ethylbenzothiazole-6-sulfonic acid
DPPH	2,2-diphenyl-1-picryhydrazyl
TAC	Total anthocyanins content
TC	Total carotenoids
TFC	Total flavonoid content
TPC	Total phenolic content
